# Lactogenic hormones alter cellular and extracellular microRNA expression in bovine mammary epithelial cell culture

**DOI:** 10.1186/s40104-016-0068-x

**Published:** 2016-02-17

**Authors:** Susumu Muroya, Tatsuro Hagi, Ataru Kimura, Hisashi Aso, Masatoshi Matsuzaki, Masaru Nomura

**Affiliations:** Animal Products Research Division, NARO Institute of Livestock and Grassland Science, Tsukuba, Ibaraki 305-0901 Japan; Faculty of Agriculture and Life Science, Hirosaki University, Hirosaki, Aomori Japan; International Education and Research Center for Food and Agricultural Immunology (CFAI), Graduate School of Agricultural Science, Tohoku University, Aoba Sendai, Japan

**Keywords:** Bovine mammary epithelial cell, Lactogenic differentiation, microRNA, Milk, Secretion

## Abstract

**Background:**

Bovine milk contains not only a variety of nutritional ingredients but also microRNAs (miRNAs) that are thought to be secreted by the bovine mammary epithelial cells (BMECs). The objective of this study was to elucidate the production of milk-related miRNAs in BMECs under the influence of lactogenic hormones.

**Results:**

According to a microarray result of milk exosomal miRNAs prior to cellular analyses, a total of 257 miRNAs were detected in a Holstein cow milk. Of these, 18 major miRNAs of interest in the milk were selected for an expression analysis in BMEC culture that was treated with or without dexamethasone, insulin, and prolactin (DIP) to induce a lactogenic differentiation. Quantitative polymerase chain reaction (qPCR) results showed that the expressions of miR-21–5p (*P* = 0.005), miR-26a (*P* = 0.016), and miR-320a (*P* = 0.011) were lower in the DIP-treated cells than in the untreated cells. In contrast, the expression of miR-339a (*P* = 0.017) in the cell culture medium were lower in the DIP-treated culture than in the untreated culture. Intriguingly, the miR-148a expression in cell culture medium was elevated by DIP treatment of BMEC culture (*P* = 0.018). The medium-to-cell expression ratios of miR-103 (*P* = 0.025), miR-148a (*P* < 0.001), and miR-223 (*P* = 0.013) were elevated in the DIP-treated BMECs, suggesting that the lactogenic differentiation-induced secretion of these three miRNAs in BMECs. A bioinformatic analysis showed that the miRNAs down-regulated in the BMECs were associated with the suppression of genes related to transcriptional regulation, protein phosphorylation, and tube development.

**Conclusion:**

The results suggest that the miRNAs changed by lactogenic hormones are associated with milk protein synthesis, and mammary gland development and maturation. The elevated miR-148a level in DIP-treated BMECs may be associated with its increase in milk during the lactation period of cows.

## Background

The mammary gland is a complex organ where the epithelial cell proliferates and differentiates during puberty, pregnancy, and lactation under the influence of various hormones such as estrogen and prolactin [1]. During pregnancy, the complexity of the ductal system increases through the addition of side branches, the formation of lobuloalveolar structures, and the differentiation of secretory epithelia. These phases of proliferation, structural formation, and lactogenic differentiation are essential to form a functional lactating mammary gland during pregnancy. The differentiation phase of mammary epithelial cells is especially important as a step to form a system for the generation and secretion of fatty acids, proteins such as caseins, and the other components of milk.

MicroRNAs (miRNAs) are highly conserved noncoding small RNAs that regulate the expression of target genes in various biological processes. Primary transcripts (pri-miRNAs) are processed into pre-miRNAs and finally into mature miRNAs that recognize target genes as components of the RNA-induced silencing complex (RISC), resulting in mRNA degradation or destabilization. In recent years, numerous studies have shown that the milk of humans, pigs, goats, cows, and mice is enriched with miRNAs [2–4], most of which are packed in extracellular microvesicles that are 30–100 nm in diameter, namely exosomes [3, 4]. Although the function of exosomal miRNAs in milk remains unknown thus far, the miRNA profile differs during lactation and between colostrum and milk in various mammal species [2, 5]. In particular, the content of miR-148a in bovine milk is elevated during 5 months of lactation [2, 3], whereas those of let-7a, miR-25, miR-30d, miR-182, miR-191, miR-200c, and miR-375 are reduced within the first month of lactation [2].

The miRNA profile in mammary gland tissue (MGT) is also affected by lactating stages [6–11] as well as by bacterial infection [12–14], indicating that a milk miRNA profile is susceptible to physiological conditions. Similarly to various milk components such as fatty acids and milk proteins including caseins, miRNAs are thought to be generated and secreted in mammary epithelial cells in mammary glands (MGs), although the quantitative ratio of MG-originated miRNA to circulation-originated miRNA remains unknown. A recent transcriptomic study using a next-generation sequencing (NGS) has revealed the miRNA expression profile of differentiated bovine mammary epithelial cells (BMECs) [15].

In addition, the expression of miR-200a is up-regulated during the differentiation of mouse mammary gland epithelial cells [16]. miR-103 is up-regulated in goat mammary glands toward mid-lactation and plays an important role in milk fat synthesis in goat mammary epithelial cells [17], suggesting that miRNAs are deeply associated with the differentiation and function of mammary epithelial cells.

It was also demonstrated recently that miR-200c in bovine milk is taken up not only by human macrophages in vitro [18] but also into human plasma [19]. Moreover, a number of miRNA species in milk including the miR-200 family are associated with immune-related function [4]. Exosomal miRNAs in milk are resistant to RNase, acidic pH, boiling, and freeze-thaw cycles to a certain extent [4, 20]. It is therefore tempting to speculate that miRNAs are transferred from milk to the recipient and then potentially function in animal development and the immune system. However, it remains unclear thus far how miRNAs in milk are generated and secreted in mammary epithelial cells including BMECs.

Here we hypothesized that major bovine milk miRNAs could be generated and secreted from BMEC in association with the lactogenic differentiation. The objective of the present study was to elucidate the generation and secretion of milk miRNAs in BMECs. To this end, we investigated intra- and extracellular expression of milk-related miRNAs in BMEC cell cultures and the alterations by lactogenesis-inducible DIP treatment using a cell line [21]. Potential molecular events with which the predicted target genes of miRNAs are associated are also discussed in light of the results of our bioinformatic analysis.

## Methods

### Milk sample preparation

The animals were cared for as outlined in the Guide for the Care and Use of Experimental Animals (Animal Care Committee of the NARO Institute of Livestock and Grassland Science), which the committee accepted. Milk samples were collected from two primiparous and three multiparous Holstein cattle (milk yield: 18.9–32.6 kg/d, days in milk: 60–112) at NARO Institute of Livestock and Grassland Science (Japan), using a clean tandem milking parlor as described [22]. To avoid mastitic milk, composite milk with low somatic cell counts (SCCs; <100,000 cells/mL) from noninflamed four quarters was selected [23]. The milk samples were immediately stored at 4 °C overnight. After centrifugation of the mixed milk samples from five cows at 2,000 *g* for 10 min at room temperature, the upper layer of fat was removed from the sample and the whey fraction was recovered. The whey sample was then centrifuged at 10,000 *g* at room temperature first for 30 min, then for 10 min, and stored at −80 °C until use. One mL of the supernatant was used for milk exosome preparation using the Total Exosome Isolation (from other body fluids) kit (Life Technologies, Tokyo) according to the manufacturer’s protocol. The final precipitate was used downstream as a milk exosome sample, which could give an averaged exosomal miRNA profile of five cows’ milk.

### BMEC culture and sample collection of cell and culture media

BMECs established as a clone [21] were cultured in 12-well plates (Life Technologies) in Dulbecco’s modified Eagle’ medium (DMEM) (Sigma, St. Louis, MO, USA) supplemented with 20 % Exo-FBS Exosome-Depleted fetal bovine serum (FBS) (System Biosciences, Mountain View, CA), 10 μg/mL apotransferrin (Sigma), 5 mM sodium acetate, 50 U/mL penicillin and 50 μg/mL streptomycin at 37 °C in 5 % CO_2_ for 7 d until the cells were confluent (approx. 2 × 10^5^ cells/well). Confluent cells were incubated for 6 d in 20 % FBS DMEM with or without lactogenic hormones consisting of 10 μg/ml dexamethasone (Sigma), 10 μg/mL bovine insulin (Sigma) and 10 μg/mL sheep prolactin (Sigma) with medium renewal at every second day. Under this condition, a cell line of bovine mammary epithelial cells differentiate and express lactogenic markers such as α-casein and α-lactalbumin in 7 d after the confluent cells are induced to differentiate [24]. Approx. 2 mL of the medium supernatant was collected as the media samples from each of three wells in a culture plate per experiment, which was repeated three times. A total of 4.8 mL of the media was prepared as a mixture of the three well samples. For a quantitative polymerase chain reaction (qPCR) analysis, the cells were also collected after two washes with phosphate-buffered saline (PBS), using RNA protect Cell Reagent (Qiagen). The exosome samples in the culture media were then centrifugally collected using an ExoQuick-TC kit (System Bioscience).

### Microarray analysis of milk exosome miRNA

For the microarray analysis of milk exosomal miRNAs, we extracted total RNA including miRNA from the samples using the mirVana™ miRNA isolation kit (Life Technologies), and determined the RNA quantity and quality of the samples using an Experion™ automated electrophoresis system with an RNA StdSens kit (Bio-Rad, Hercules, CA). The RNA sample of exosomes from mixed milk of five cows was applied to an Affymetrix GeneChip® miRNA 4.0 Array (Affymetrix, Santa Clara, CA) that corresponds to miRBase ver.20 and comprehensively covers 203 organisms including bovine (http://www.affymetrix.com/estore/catalog/131473/AFFY/miRNA+Array#1_1). The signals of hybridized probes in the array were scanned with a GeneChip® Scanner 3000 7G (Affymetrix). The scanned microarray data were analyzed with Affymetrix® Expression Console™ Software (Affymetrix).

### RNA preparation and cDNA synthesis of cell and the culture media samples for qPCR

For miRNA qPCR analysis, total RNA including miRNA was extracted from the exosomes of cells and the culture media samples, using the mirVana™ miRNA isolation kit (Life Technologies). cDNA for the qPCR of miRNAs was synthesized from 250 ng of total RNA for the cell samples or from 9 μL of the final product of RNA preparation for the culture medium samples, using the miScript II RT kit (Qiagen) at 37 °C for 60 min, and then the enzyme was inactivated at 95 °C for 5 min.

For qPCR analysis of mRNA in cells, total RNA excluding miRNA was extracted using the RNeasy Plus Mini Kit (Qiagen, Tokyo) and an RNase-Free DNase Set (Qiagen). cDNA was synthesized from 1,000 ng of the total RNA using a PrimeScript® II 1st strand cDNA Synthesis Kit (Takara, Otsu, Japan). The resulting cDNA solutions were diluted with sterile distilled water and used as templates for qPCR.

### Quantitative PCR (qPCR) analysis

The miRNA qPCR was performed using a CFX96 thermal cycler (Bio-Rad) under the following program: first for 15 min at 95 °C, followed by 40 cycles of 15 s at 95 °C and 30 s at 60 °C, with the Thunderbird SYBR qPCR kit (Toyobo, Tokyo) in combination with the miScript Primer Assay for let-7b, miR-21-5p, miR-23b-3p, miR-25, miR-26a, miR-30a, miR-103, miR-107, miR-148a, miR-155, miR-182, miR-191, miR-200c, miR-221, miR-223, miR-320a, miR-339a, and miR-375 (Qiagen). Cellular RNU6-6P RNA (RNU6-6P) and exogenous cel-miR-39 (Qiagen) were used as an internal control for cell samples and as a spike-in control for medium samples, respectively. The resulting values of qPCR for cellular and medium miRNAs were normalized by those of RNU6-6P and cel-miR-39, respectively. The results of miRNAs were quantified using respective standard curves.

A qPCR of GAPDH, β- and κ-casein was performed using the Thunderbird SYBR qPCR Mix (Toyobo) for BMEC cell samples with ribosomal RNA 18 s (R18s) as an internal control. The thermal cycling conditions used were: 95 °C for 10 s, followed by 40 cycles of 95 °C for 5 s and 62 °C for 30 s. The melting program was 95 °C for 10 s, 65 °C for 5 s and 95 °C for 50 s. A melting curve analysis was used to confirm the specificity of the amplification. The PCR primers used in this study were as shown in Table 1 [24, 25]. Fold changes were determined by the threshold cycle (Ct). Fold changes of miRNA expression were calculated using the 2^−ΔCt^ method, where ΔCt = (Ct _target_ − Ct _control_) _Sample_ [7].

**Table 1:** PCR primers used in this study

Target gene		Sequence
*GAPDH*	fw	5’-GGGTCATCATCTCTGCACCT-3’
	rv	5’-GGTCATAAGTCCCTCCACGA-3’
*β-Casein*	fw	5’-GTGAGGAACAGCAGCAAACA-3’
	rv	5’-TTTTGTGGGAGGCTGTTAGG-3’
*κ-Casein*	fw	5’-CCAGGAGCAAAACCAAGAAC-3’
	rv	5’-TGCAACTGGTTTCTGTTGGT-3’
*R18S*	fw	5’-CGGGGAGGTAGTGACGAAA-3’
	rv	5’-CCGCTCCCAAGATCCAACTA-3’

### Prediction and functional annotation of miRNA target genes

The bioinformatic analysis was conducted as described [21]. In brief, the miRNA target genes were predicted using the TargetScan (Release 6.2, http://www.targetscan.org/) [26]. The miRNA sequences applied to the analyses were of bovines. To classify the target genes according to the functional annotation, we conducted a gene ontology (GO) analysis on the target genes of differentially expressed miRNAs between treatments with dexamethasone, insulin, and prolactin (DIP). In this study, the Database for Annotation, Visualization, and Integrated Discovery (DAVID) bioinformatic resources (version 6.7, http://david.abcc.ncifcrf.gov) [27] were applied to the potential target genes with the setting *bos taurus* (domestic cow) as the background species, to enrich characteristic GO terms for the respective miRNA-mediated biological process. Extraction of the terms was considered significant when the Bonferroni probability value (*P*_B_) was < 0.05.

### Statistical analysis

The expression data are shown as means ± SD and were compared by statistical analyses with a significance level of *P* < 0.05. The comparisons between with and without DIP treatment were carried out by the two-sided Student’s *t*-test, using js-STAR 2012 software (ver. 2.0.6j; http://www.kisnet.or.jp/nappa/software/star/index.htm).

## Results

### Composition of exosomal miRNAs in milk

**Fig. 1:**
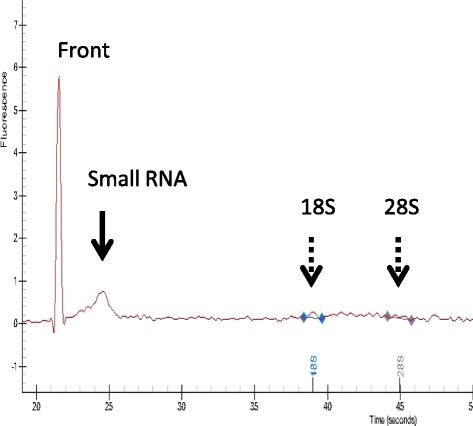
Representative electropherogram of RNA samples prepared from milk exosomes of Holstein cows. The peaks of ribosomal-18 s (18 s) and -28 s (28 s) RNA were at only a trace level compared to that of small RNA

**Fig. 2:**
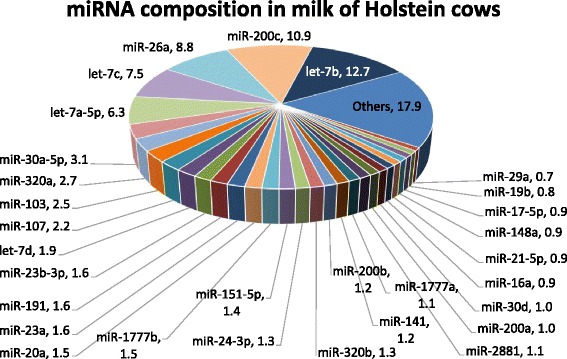
The microRNA composition in milk exosomes of Holstein cows. The microRNA (miRNA) names and the percentages of total miRNA contents are indicated

According to the qualitative analysis of milk exosomal RNAs, most of the RNAs composing milk exosomes were small RNAs including miRNAs (Fig. 1). The results of our microarray analysis showed that, of 783 bovine miRNAs registered in the miRBase (ver. 20), a total of 257 miRNAs were detected in the milk exosomes. The top 20 miRNAs in the milk exosome were let-7b (12.7 %), miR-200c (10.9 %), miR-26a (8.8 %), let-7c (7.5 %), let-7a-5p (6.3 %), miR-30a-5p (3.1 %), miR-320a (2.7 %), miR-103 (2.5 %), miR-107 (2.2 %), let-7d (1.9 %), miR-23-3p (1.6 %), miR-191 (1.6 %), miR-23a (1.6 %), miR-20a (1.5 %), miR-1777b (1.5 %), miR-151-5p (1.4 %), miR-24-3p (1.3 %), miR-320b (1.3 %), miR-200b (1.2 %), and miR-141 (1.2 %) (Fig. 2).

### MicroRNA expression in DIP-treated BMECs and the culture medium

**Fig. 3:**
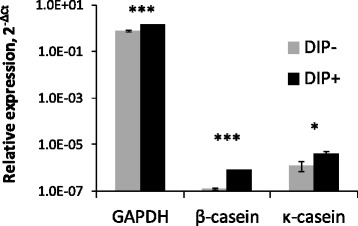
Quantitative PCR results of GAPDH and lactogenic markers (β- and κ-caseins) in differentiated and undifferentiated BMECs. *P < 0.05, *** *P* < 0.001 indicate the differences between the BMEC cultures with (DIP+) and without lactogenic hormones (DIP−)

We investigated the changes in the generation of milk-abundant miRNAs in BMECs during the DIP-induced differentiation process. It has been shown that lactogenic differentiation can be induced by addition of DIP to BMEC culture [21, 28]. All FBS used was from a single lot, to ensure that any effect of serum was consistent between treatments. No significant difference in expression level of RNU6-6P or cel-miR-39 was observed between DIP-treated and untreated cells or the culture media. When BMECs were treated with DIP, the expressions of GAPDH and the lactogenic differentiation markers, namely β- and κ-casein, were elevated compared to those of the untreated BMECs (*P* < 0.001, *P* < 0.001, and *P* = 0.013, respectively) (Fig. 3), indicating that the BMECs were induced to lactogenic differentiation.

**Fig. 4:**
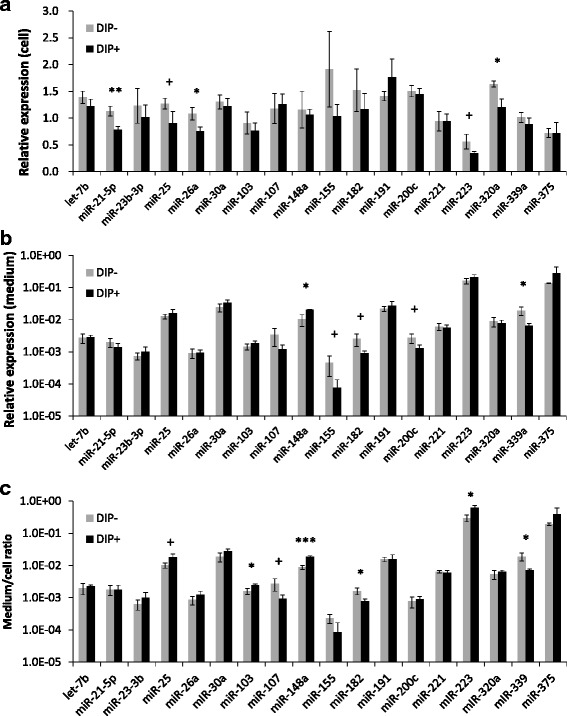
Quantitative PCR results of miRNAs in differentiated and undifferentiated BMECs. **a**: Cellular miRNAs of BMECs. **b**: miRNAs in BMEC culture media. **c**: The miRNA expression ratio of medium/cells. ^+^ *P* < 0.10; * *P* < 0.05, ** *P* < 0.01, and *** *P* < 0.001 indicate differences between BMEC cultures with (DIP+) and without lactogenic hormones (DIP−) for each of the miRNAs

In the DIP-treatment of BMECs, both the cellular and extracellular expression of miRNAs were analyzed. The cellular expression of miR-21-5p, miR-25, miR-26a, miR-223, and miR-320a was lower in the DIP-treated BMECs than in the untreated BMECs (*P* = 0.005, = 0.059, = 0.016, = 0.054, and = 0.011, respectively), whereas that of the other miRNAs was not significantly changed by the treatment (Fig. 4a). In contrast, the expressions of miR-155, miR-182, miR-200c, and miR-339a in the BMEC culture medium were lower in the DIP-treated BMECs than in the untreated BMECs (*P* = 0.088, = 0.061, = 0.067, and = 0.017, respectively) (Fig. 4b). In addition, it is especially unique that the miR-148a expression in the medium was up-regulated in the DIP-treated BMECs compared to that in the untreated cells (*P* = 0.013).

We also estimated the ratio of miRNA in the medium to that in the cells, to clarify the influence of differentiation on the miRNA distribution between the outside and the inside of the BMECs. The ratios of miR-25, miR-103, miR-148a, and miR-223 were elevated (*P* = 0.062, = 0.025, < 0.001, and = 0.014, respectively) but those of miR-107, miR-182, and miR-339a were reduced in the DIP-treated BMECs (*P* = 0.057, = 0.022, and = 0.020, respectively) in comparison to those in the untreated cells (Fig. 4c). Intriguingly, among the miRNAs tested, the elevated miR-148a expression and the reduced miR-339a expression in the BMEC culture media were consistent with the expression ratio of the medium to the cells, indicating that both the biogenesis and the secretion of those miRNAs were changed cooperatively.

### Potential molecular events associated with microRNAs that changed with DIP-treatment of BMEC

We further analyzed the predicted target genes of the relevant miRNAs by using the functional annotation in DAVID, to analyze the molecular events associated with miRNAs that are affected by DIP-treatment in BMECs. According to the results of our TargetScan analysis, a total of 1,617 bovine genes are predicted as the targets of significantly reduced miRNAs by DIP-treatment of BMEC (miR-21-5p, miR-26a, and miR-320a). Of those target genes, 1,382 genes identified in the DAVID program were further applied to the functional annotation analysis, which resulted in the extraction of the over-represented GO terms such as the regulation of transcription, phosphorylation, the regulation of macromolecule metabolism, and signaling pathways related to small GTP-binding protein and enzyme-linked receptor (*P*_B_ < 0.05, Table 2).

**Table 2:** Gene ontology terms enriched with predicted miR-21-5p, miR-26a, and miR-320a target genes

Term	Target genes	Bonferroni
GO:0045449 ~ regulation of transcription	134	0.00001
GO:0051252 ~ regulation of RNA metabolic process	105	0.00002
GO:0006468 ~ protein amino acid phosphorylation	66	0.00002
GO:0006355 ~ regulation of transcription, DNA-dependent	102	0.00007
GO:0006796 ~ phosphate metabolic process	82	0.00026
GO:0006793 ~ phosphorus metabolic process	82	0.00026
GO:0016310 ~ phosphorylation	69	0.00068
GO:0048598 ~ embryonic morphogenesis	26	0.00141
GO:0007242 ~ intracellular signaling cascade	65	0.00269
GO:0010604 ~ positive regulation of macromolecule metabolic process	46	0.00288
GO:0007167 ~ enzyme linked receptor protein signaling pathway	27	0.00632
GO:0035295 ~ tube development	22	0.00687
GO:0010628 ~ positive regulation of gene expression	35	0.01255
GO:0007264 ~ small GTPase mediated signal transduction	30	0.01361
GO:0051173 ~ positive regulation of nitrogen compound metabolic process	37	0.01550
GO:0031328 ~ positive regulation of cellular biosynthetic process	39	0.01838
GO:0045935 ~ positive regulation of nucleobase, nucleoside, nucleotide and nucleic acid metabolic process	36	0.01985
GO:0009891 ~ positive regulation of biosynthetic process	39	0.02411
GO:0045941 ~ positive regulation of transcription	33	0.03073
GO:0019941 ~ modification-dependent protein catabolic process	35	0.03719
GO:0043632 ~ modification-dependent macromolecule catabolic process	35	0.03719
GO:0010557 ~ positive regulation of macromolecule biosynthetic process	37	0.03929
GO:0006357 ~ regulation of transcription from RNA polymerase II promoter	37	0.04292

The miR-148a was unique in that its expression in BMEC culture medium was elevated by DIP-treatment of the cells. A total of 630 bovine genes are predicted as the targets of miR-148a. Of those target genes, we further analyzed 530 genes identified in the DAVID program by functional annotation, which extracted relevant GO terms such as the regulation of transcription, phosphorylation, the regulation of macromolecule metabolism, and blood vessel development (*P*_B_ < 0.05, Table 3). Regarding miR-339a, which showed significant down-regulation of its expression in the BMEC culture medium, the results of the TargetScan analysis predicted a total of 177 genes including MyoD, a master regulator of myogenesis, and B-cell CLL/lymphoma 6 (BCL6) as the targets of miR-339a. However, none of the significant molecular biological terms were extracted by the GO analysis using the miR-339a target genes.

**Table 3:** Gene ontology terms enriched with predicted miR-148a target genes

Term	Target genes	Bonferroni
GO:0045449 ~ regulation of transcription	67	0.00044
GO:0010628 ~ positive regulation of gene expression	23	0.00096
GO:0051252 ~ regulation of RNA metabolic process	53	0.00159
GO:0045941 ~ positive regulation of transcription	22	0.00171
GO:0006355 ~ regulation of transcription, DNA-dependent	52	0.00218
GO:0045935 ~ positive regulation of nucleobase, nucleoside, nucleotide and nucleic acid metabolic process	23	0.00280
GO:0001568 ~ blood vessel development	16	0.00430
GO:0051173 ~ positive regulation of nitrogen compound metabolic process	23	0.00462
GO:0001944 ~ vasculature development	16	0.00591
GO:0031328 ~ positive regulation of cellular biosynthetic process	23	0.01828
GO:0009891 ~ positive regulation of biosynthetic process	23	0.02192
GO:0010604 ~ positive regulation of macromolecule metabolic process	25	0.02990
GO:0010557 ~ positive regulation of macromolecule biosynthetic process	22	0.03007
GO:0001525 ~ angiogenesis	11	0.04250
GO:0006796 ~ phosphate metabolic process	40	0.04491
GO:0006793 ~ phosphorus metabolic process	40	0.04491

Moreover, we also conducted bioinformatics analysis on the relevant miRNAs, miR-103, miR-148a, and miR-223, which showed an elevation of the medium/cell ratios. Of the 1490 potential target genes of those miRNAs, a total of 1121 bovine genes were used as valid genes. The result of GO analysis indicated that the target genes are associated with transcriptional regulation of gene expression, post-translational modification, and blood vessel formation (Table 4).

**Table 4:** Gene ontology terms enriched with predicted miR-103, miR-148a, and miR-223 target genes

Term	Target genes	Bonferroni
GO:0045449 ~ regulation of transcription	127	1.51E-08
GO:0051252 ~ regulation of RNA metabolic process	100	1.43E-07
GO:0006355 ~ regulation of transcription, DNA-dependent	97	6.19E-07
GO:0045941 ~ positive regulation of transcription	37	9.14E-06
GO:0010628 ~ positive regulation of gene expression	38	9.83E-06
GO:0006468 ~ protein amino acid phosphorylation	60	1.34E-05
GO:0045935 ~ positive regulation of nucleobase, nucleoside, nucleotide and nucleic acid metabolic process	39	1.72E-05
GO:0006796 ~ phosphate metabolic process	76	2.98E-05
GO:0006793 ~ phosphorus metabolic process	76	2.98E-05
GO:0051173 ~ positive regulation of nitrogen compound metabolic process	39	4.02E-05
GO:0010557 ~ positive regulation of macromolecule biosynthetic process	40	4.04E-05
GO:0031328 ~ positive regulation of cellular biosynthetic process	41	4.49E-05
GO:0009891 ~ positive regulation of biosynthetic process	41	6.27E-05
GO:0010604 ~ positive regulation of macromolecule metabolic process	45	8.95E-05
GO:0001525 ~ angiogenesis	18	4.58E-04
GO:0016310 ~ phosphorylation	62	5.84E-04
GO:0001568 ~ blood vessel development	24	8.85E-04
GO:0001944 ~ vasculature development	24	0.00141236
GO:0045944 ~ positive regulation of transcription from RNA polymerase II promoter	26	0.00405696
GO:0048514 ~ blood vessel morphogenesis	20	0.01132062
GO:0045893 ~ positive regulation of transcription, DNA-dependent	27	0.02355478
GO:0051254 ~ positive regulation of RNA metabolic process	27	0.02355478
GO:0006357 ~ regulation of transcription from RNA polymerase II promoter	34	0.02446326

## Discussion

In the present study, to elucidate the generation and secretion of miRNAs in BMEC culture, we focused on a total of 18 milk-related miRNAs that were contained in milk from Holstein cows. Of those, miR-7b, miR-21-5p, miR-23-3p, miR-26a, miR-30a, miR-103, miR-107, miR-148a, miR-200c, and miR-320a were among the top 30 most abundant miRNAs in the milk. The expression levels of miR-25, miR-155, miR-182, miR-191, miR-221, miR-223, and/or miR-375 have been reported to change in the mammary gland tissues of cows [7], goats [8], milk from pigs [3], and rat milk whey [29] during lactation, and therefore we also analyzed these miRNAs.

As indicated by the elevated expression of lactogenic markers (β- and κ-caseins) [1], we concluded that the BMECs were induced into lactogenic differentiation by the addition of DIP in the present study, though milk fat and protein secretion were not determined. Nevertheless, this BMEC is able to not only express lactogenic gene mRNAs but also secret milk proteins such as α-casein by seven days after induction of differentiation with DIP treatment [21, 30]. Another BMEC under DIP treatment also can successfully differentiate in seven days of lactogenic differentiation [28], indicating that DIP treatment is able to induce functional lactogenic differentiation of BMEC *in vitro*.

The lactation stage in which milk protein is acceleratingly secreted is defined as ‘secretory activation’ [31], the third stage of four functional differentiation stages of the mammary gland [1]. In mice and rats, an increase in activity of lipid synthetic enzymes was observed in the second stage [32]. This is followed by activation of the transcription of milk protein genes in the third stage [33, 34], in which prolactin is increasingly secreted from pituitary and plays a pivotal role in initiation of lactation [1, 35]. Since an increase in β- and κ- caseins expression was induced by lactogenic hormones such as prolactin in the present study, the process observed in the BMEC culture could be a model of the third stage (secretory activation) that begins at or around parturition, continuing at least to the early phase of lactation [1]. The elevated expression level of GAPDH in the BMEC culture might reflect an accelerated glucose consumption to generate energy for milk component production.

We hypothesized that generation and secretion of miRNAs in BMEC could be promoted during lactogenic differentiation. Intriguingly, according to the miRNA qPCR results, it is likely that most of cellular miRNAs including miR-21-5p, miR-26a, and miR-320a in BMEC culture are down-regulated after DIP treatment. Thus, it is suggested that down-regulation of those miRNAs could be associated with release of translational regulation of lactogenic genes in BMEC. The expression level of miR-21-5p was reported to be up-regulated in bovine MGT at 7 d postpartum compared to −30 d prepartum [9], whereas its level in porcine milk analyzed by qPCR was down-regulated at 7–14 d of the lactation period from day 0 but was followed by a marked increase at the 21 d [3]. In addition, miR-21-5p was at a constant level in rat whey [29], and changed both upward and downward during lactation [2]. Thus, miR-21-5p expression may be affected by period of lactation, species, and/or differences in cell behavior between in vivo and in vitro.

The expression level of miR-26a in bovine MGT was down-regulated during lactation [7], which supports the present finding of a down-regulation of miR-26a in BMEC treated with DIP. The reduced expression of miR-26a in BMECs during differentiation could account for that in bovine MGT during lactation.

Bioinformatic analysis using target genes of miR-21-5p, miR-26a, and miR-320a predicted that the molecular biological events with which the target genes of those miRNAs were associated are transcriptional regulation, protein phosphorylation, phosphate metabolic process, embryonic morphogenesis, tube development, and positive regulation of biosynthetic process. All of these predicted molecular biological events could be associated with the formation of a ductal and lobulo-aveolar structure and with the acceleration of the synthesis of milk ingredients during the secretory differentiation of mammary glands and lactation. These terms of biological process suggest that the down-regulations of miR-21-5p, miR-26a, and miR-320a in BMECs may have roles in the mammary gland differentiation and lactation of dairy cows.

We also observed that the expression level of miR-148a in the culture medium of DIP-treated BMEC was higher than that in the untreated cell culture medium in the present study, although its expression in the cells did not change with DIP treatment. Intriguingly, miR-148a is unique in that its elevated expression in milk and MGT during lactation has been consistently reported [2, 3, 7]. In addition, the medium/cell ratio of miR-148a expression was up-regulated with induction of lactogenic differentiation in BMECs in this study. These results suggest that the increase in miR-148a expression in milk during lactation may be due to the elevated secretion of miR-148a by differentiated mammary epithelial cells. As well as miR-148a, miR-103 and miR-223 expression in BMEC culture media was also elevated by DIP-treatment, suggesting secretion of miR-103 and miR-223 in BMEC was also enhanced by lactogenic hormones.

The role of miR-148a secreted in milk remains unknown, however. It was reported that miR-148a promotes myogenic differentiation by targeting the Rho-associated coiled-coil containing protein kinase 1 (ROCK1) gene [36]. In addition, miR-148a is associated with osteogenic differentiation [37] and angiogenesis in breast cancer by targeting not only v-erb-b2 erythroblastic leukemia viral oncogene homolog 3 (ERBB3) [38] but also DNA methyltransferase-1 (DNMT1), insulin-like growth factor-1 receptor (IGF-1R), and insulin receptor substrate-1 (IRS-1) [39]. Extracellular miRNAs secreted into body fluids such as milk and blood are used in cell–cell communication [40, 41]; for example, colostrum-derived miRNAs are taken up in vitro by macrophages and modulate their immune activities, such as migration and cytokine secretion [42]. The miR-148a that is secreted by BMECs into milk may thus affect its target gene expression in the recipient cells of the secreted miRNA, such as that in muscles and bones [43]. Bioinformatic analysis of miR-148a resulted in the extraction of GO terms associated with the regulation of transcription, angiogenesis, and the phosphate metabolic process. The genes related to these events in potential recipient cells of miR-148a may also be affected by the miR-148a in milk.

The expression level of miR-339a in the BMEC culture medium was reduced by DIP treatment in this study. The known role of miR-339a is limited to cancer-related functions such as the down-regulation of BCL6 expression [44]. BCL6 is essential for the promotion of mammary epithelial cell survival [45]. In the present study, even though significant molecular events were not extracted by the GO analysis, MyoD and BCL6 were predicted as the targets. Although the biological function of miR-339a in milk remains unknown, its reduced expression in the BMEC culture medium may indicate a release of the suppression of the target gene expression in the exosome recipient cells in skeletal muscle and mammary gland tissues.

## Conclusion

We investigated changes in both cellular and extracellular miRNAs extracted from BMEC in cell culture under the influence of lactogenic hormones. In the qPCR analyses of 18 miRNAs that were abundant in milk, the expressions of miR-21-5p, miR-26a, and miR-320a were significantly lower in the DIP-treated cells than in the untreated cells. In a cell culture medium, miR-339a expression was lower and miR-148a expression was higher in the DIP-treated culture than in the untreated culture. The results of a bioinformatic analysis suggested that the miRNAs down-regulated in the BMECs were associated with molecular biological events that are essential to mammary gland development and maturation. The elevated miR-148a level during BMEC differentiation may be associated with its increase in milk during the lactation period of cows, though secretion of milk proteins is not determined in the present study.
